# Analysis of the complete organellar genomes of the economically valuable kelp *Lessonia spicata* (Lessoniaceae, Phaeophyceae) from Chile

**DOI:** 10.1080/23802359.2019.1640647

**Published:** 2019-07-16

**Authors:** Daniel Tineo, Karol B. Rubio, Jegnes B. Melendez, Jani E. Mendoza, Jhonsy O. Silva, Jhordy Perez, Eggleantina E. Esquerre, Melissa Perez-Alania, Samia L. Fernandez, Smith E. Aguilar, Fernando Chuquizuta, Yadira M. Olano, Renzo P. Hoyos, Jaris E. Veneros, Ligia M. Garcia, Natalia Arakaki, Enrique Garcia-Candela, Manuel Oliva, Andres Mansilla, Martha S. Calderon, Jeffery R. Hughey, Danilo E. Bustamante

**Affiliations:** a Instituto de Investigación para el Desarrollo Sustentable de Ceja de Selva (INDES-CES), Universidad Nacional Toribio Rodríguez de Mendoza, Amazonas, Peru;; b Facultad de Ciencias, Universidad Nacional Agraria La Molina, Lima, Peru;; c Facultad Ingeniería Zootecnista, Agronegocios y Biotecnología (FIZAB), Universidad Nacional Toribio Rodríguez de Mendoza, Amazonas, Peru;; d Facultad de Ingeniería Civil y Ambiental (FICIAM), Universidad Nacional Toribio Rodríguez de Mendoza, Amazonas, Peru;; e Facultad de Ingeniería y Ciencias Agrarias (FICA), Universidad Nacional Toribio Rodríguez de Mendoza, Amazonas, Peru;; f Banco de Germoplasma de Organismos Acuáticos, Instituto del Mar del Perú, Callao, Peru;; g Instituto Tecnológico de la Producción, CITEacuícola Ahuashiyacu, San Martin, Peru;; h Laboratorio de Ecosistemas Marinos Antárticos y Sub-antárticos (LEMAS), Universidad de Magallanes, Punta Arenas, Chile;; i Division of Mathematics, Science, and Engineering, Hartnell College, Salinas, CA, USA

**Keywords:** Chile, kelp, *Lessonia*, mitogenome, plastid genome

## Abstract

*Lessonia spicata* (Suhr) Santelices is the most ecologically and economically important kelp from Pacific South America. Here, we contribute to the bioinformatics and evolutionary systematics of the species by performing high throughput sequencing on *L. spicata* from Valparaiso, Chile. The *L. spicata* complete mitogenome is 37,097 base pairs (bp) in length and contains 66 genes (GenBank accession MK965907), the complete plastid genome is 130,305 bp and has 173 genes (accession MK965908), and the data assembled 7,630 bp of the nuclear ribosomal cistron (accession MK965909). The organellar genomes are similar in structure and content to others published from the Laminariales.

*Lessonia spicata* is a common intertidal to shallow subtidal kelp distributed from central (29° S) to southern (41° S) Chile (González et al. 2012). This species is characterized as having a dichotomous stipe with longitudinal splits and numerous branches of the thallus, with each branch with a single, narrow, terminal blade (von Suhr 1839; Searles 1978). *Lessonia spicata* is considered the most ecologically important and dominant seaweed on the Pacific South American coast (Santelices et al. 1980; González et al. 2012). It is also economically valuable and is harvested for the phycocolloid alginate (Díaz et al. 2012). To contribute to the evolutionary systematics of the Laminariales and to advance the understanding of the taxonomy of *L. spicata*, this study characterized the complete organellar genomes and the nuclear ribosomal cistron of *L. spicata* from Reñaca beach, Valparaiso, Chile.

DNA was extracted from *L. spicata* (Specimen Voucher-DBM0003) using the Quick-DNA Plant/Seed kit (Zymo Research, California, USA) following the manufacturer’s instructions. The 150 bp PE Illumina library construction and sequencing was performed using myGenomics, LLC (Alpharetta, Georgia, USA). The genomes were assembled using default de novo settings in CLC Genomics Workbench 12.0 (QIAGEN Bioinformatics, Redwood City, CA, USA) and Geneious Prime to close gaps (Biomatters, Ltd, Auckland, New Zealand). The genes were annotated manually using blastx, NCBI ORFfinder, and tRNAscan-SE 1.21 (Schattner et al. 2005). The *L. spicata* mitogenome was aligned to other mitogenomes using MAFFT (Katoh and Standley 2013). The phylogenetic analysis was executed with RAxML-NG (Kozlov et al. 2018) using the GTR + gamma model and 1000 bootstraps. The tree was visualized with TreeDyn 198.3 at Phylogeny.fr (Dereeper et al. 2008).

The mitogenome of *L. spicata* is 37,097 bp in length and contains 66 genes. It is A + T rich (67.3%) and includes 25 tRNA (trnK and trnS occur in duplicate, trnL and trnM in triplicate), 17 ribosomal proteins, three rRNA (rnl, rns, rrn5), three orfs (orf41, orf129, orf378), and 18 other genes involved in electron transport and oxidative phosphorylation. The plastid genome of *L. spicata* is 130,305 bp and contains 173 genes. It is A + T biased (69.1%) and includes 45 ribosomal proteins, 27 tRNA (trnA, trnG, trnI, trnR, and trnS occur in duplicate, trnM occurs in triplicate), 27 photosystem I and II, 20 ycf, eight cytochrome b/f complex, eight ATP synthase, four RNA polymerase, six rRNA, and 28 other genes. The mitogenome and plastid genome of *L. spicata* are similar in length, content, and organization to other Laminariales (Oudot-le secq et al. 2006; Yotsukura et al. 2010; Li et al. 2015; Qu et al. 2015; Zhang et al. 2015; Chen et al. 2019; Zheng et al. 2019).

Phylogenetic analysis of the *L. spicata* mitogenome positions it in a clade with *Laminaria digitata* and *L. hyperborea* (Figure 1). This evolutionary relationship is similar to the most recent multigene and transcriptome analyses of the Laminariales in which the Lessoniaceae is closely allied with the Laminariaceae (Kawai 2014; Kawai et al. 2017; Starko et al. 2019).

**Figure 1.**
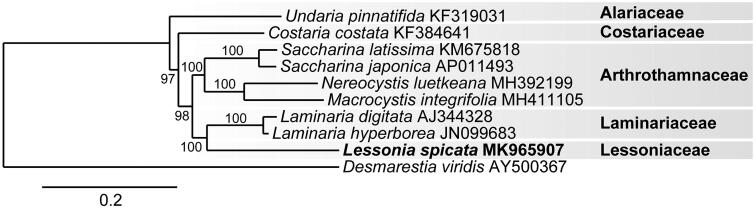
Maximum likelihood phylogram of *Lessonia spicata* (MK965907) and related Laminarialean mitogenomes. Numbers along branches are RaxML bootstrap supports based on 1000 replicates. The legend below represents the scale for nucleotide substitutions.
